# Comment on ‘Effectiveness and safety of central pancreatectomy in benign or low-grade malignant pancreatic body lesions: a systematic review and meta-analysis’

**DOI:** 10.1097/JS9.0000000000001100

**Published:** 2024-01-18

**Authors:** Hsiu-Lan Weng, Ying-Jen Chang, Ming Yew, Kuo-Chuan Hung

**Affiliations:** a Department of Anesthesiology, E-Da Hospital, I-Shou University, Kaohsiung City; b Department of Anesthesiology, Chi Mei Medical Center, Tainan City, Taiwan

*Dear Editor*,

We read with great interest the systematic review and meta-analysis by Bi *et al*.^1^ comparing the safety and efficacy of central pancreatectomy (CP) versus distal pancreatectomy (DP) for benign or low-grade malignant pancreatic body lesions. Their meta-analysis found that CP was associated with longer operative times, greater blood loss, and higher rates of most postoperative complications, including pancreatic fistula, hemorrhage, morbidity, and longer hospital stays than DP^1^. However, CP better preserved endocrine and exocrine functions in the long term. This is an important topic as surgeons continue to debate the appropriate pancreatic parenchymal-sparing operation for small, low-grade pancreatic neck/body lesions. The present investigation included 26 studies comprising nearly 2500 patients, thereby constituting the most extensive examination of the subject to date^1^. The authors are worthy of commendations on the comprehensive analyses.

Nevertheless, the presence of significant heterogeneity in meta-analyses warrants thorough investigation of its causes. In the original meta-analysis^1^, a high degree of heterogeneity was found for operative time (i.e. 90%) and blood loss when comparing CP and DP. The authors performed subgroup analyses based on the surgical approach and study design without identifying significant sources of heterogeneity^1^. Given the inclusion of studies from multiple countries across Asia, Europe, and North America, we hypothesized that geographic regions may be a source of heterogeneity. Surgical practices, training, and techniques may differ between countries in a way that affects operative times for complex pancreatic resections, such as CP. To further analyze this, we collected the raw data of the original meta-analysis^1^ and divided the included studies by country into Asian (China, Japan, and Korea) and non-Asian (the USA, Italy, Germany, Spain, France, and Romania) subgroups. Our subgroup analysis was performed using the Cochrane Review Manager (RevMan 5.3; Copenhagen: The Nordic Cochrane Center, The Cochrane Collaboration, 2014) as previously reported^2^. As shown in Figure 1, this stratification reduced the heterogeneity to 85% in the Asian subgroup (mean difference, 62.89; 95% confidence interval: 43.32–82.45; *P*<0.00001) and 75% in the non-Asian subgroup (mean difference, 27.44; 95% confidence interval: 9.8–45.09; *P*=0.002). The difference between the subgroups was statistically significant (mean difference: 62.89 vs. 27.44; *P*=0.008), indicating that the country/region of the study partially explained some of the variability in operative times.

**Figure 1:**
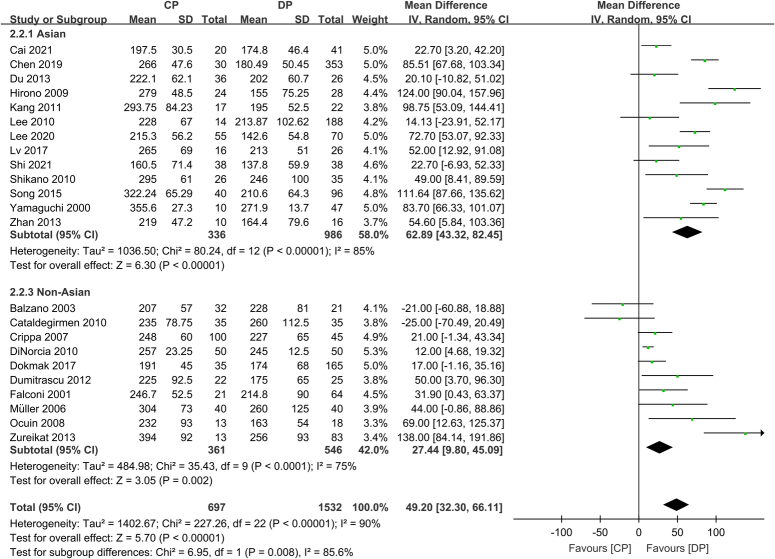
Forest plot comparing central pancreatectomy (CP) versus distal pancreatectomy (DP) on operative time in the Asian and non-Asian subgroups. The forest plot shows mean differences with 95% confidence intervals (CIs) for each study, as well as the pooled mean differences for each subgroup and overall. The diamond shapes represent the pooled effect estimate for each subgroup and overall, with their widths reflecting the precision of the pooled estimates. The test for subgroup differences showed a significant interaction (*P*=0.008), indicating that the difference between Asian and non-Asian subgroups contributes to the overall heterogeneity.

In conclusion, the significant heterogeneity in operative times between CP and DP across the included studies can be partially explained by geographic region. This analysis generated the hypothesis that operative time may differ between Asian and non-Asian countries, although the specific country-level factors contributing to this possible discrepancy require further investigation. Additional well-designed studies controlling for country and surgical approach are needed for definitive conclusions.

## Ethical approval

Not applicable.

## Consent

Not applicable.

## Sources of funding

No external funding was received for this study.

## Author contribution

H.-L.W. and K.-C.H.: wrote the main manuscript text; Y.-J.C. and M.Y.: collected data and prepared Figure 1. All authors read and approved the final version of the manuscript.

## Conflicts of interest disclosure

The authors declare no conflicts of interest.

## Research registration unique identifying number (UIN)

Not applicable.

## Guarantor

Kuo-Chuan Hung.

## Data availability statement

The datasets used and/or analyzed in the current study are available from the corresponding author upon reasonable request.

## Provenance and peer review

This paper was not invited.
